# Influence of prior pH and thermal stresses on thermal tolerance of foodborne pathogens

**DOI:** 10.1002/fsn3.1034

**Published:** 2019-05-14

**Authors:** Chyer Kim, Mariam Bushlaibi, Rana Alrefaei, Eunice Ndegwa, Paul Kaseloo, Crystal Wynn

**Affiliations:** ^1^ Agricultural Research Station Virginia State University Petersburg Virginia; ^2^ Department of Biology Virginia State University Petersburg Virginia; ^3^ Department of Family and Consumer Sciences Virginia State University Petersburg Virginia

**Keywords:** *D*‐value, pH, *Salmonella*, *Staphylococcus*, temperature, *z*‐value

## Abstract

Improper food processing is one of the major causes of foodborne illness. Accurate prediction of the thermal destruction rate of foodborne pathogens is therefore vital to ensure proper processing and food safety. When bacteria are subjected to pH and thermal stresses during growth, sublethal stresses can occur that may lead to differences in their subsequent tolerance to thermal treatment. As a preliminary study to test this concept, the current study evaluated the effect of prior pH and thermal stresses on thermal tolerance of *Salmonella* and *Staphylococcus* using a tryptic soy broth supplemented with yeast extract. Bacteria incubated at three pH values (6.0, 7.4, and 9.0) and four temperatures (15, 25, 35, and 45°C) for 24 hr were subjected to thermal treatments at 55, 60, and 65°C. At the end of each treatment time, bacterial suspensions were surface‐plated on standard method agar for quantification of bacterial survival and further calculation of the thermal death decimal reduction time (*D*‐value) and thermal destruction temperature (*z*‐value). The effect of pH stress alone during the incubation on the thermal tolerance of both bacteria was generally insignificant. An increasing pattern of *D*‐value was observed with the increment of thermal stress (incubation temperature). The bacteria incubated at 35°C required the highest *z*‐value to reduce the 90% in *D*‐values*. Staphylococcus* mostly displayed higher tolerance to thermal treatment than *Salmonella*. Although further research is needed to validate the current findings on food matrices, findings in this study clearly affirm that adaptation of bacteria to certain stresses may reduce the effectiveness of preservation procedures applied during later stage of food processing and storage.

## 1 INTRODUCTION

Knowledge of bacterial responsiveness over a range of conditions enables predictions of bacterial growth and destruction. Using this information, questions about microbial food safety may be answered by objective analysis based on scientific data. This is especially relevant in light of the continuous occurrences of food product recalls and foodborne outbreaks throughout the world (CDC, 2018; Flynn, 2018; News Desk, 2018; Whitworth, 2018). Microbial growth is greatly influenced by pH and temperature (Food and Drug Administration (FDA) 2011). Consequently, food manufacturing processes that modify either or both the pH and temperature of foods are extensively used as mechanisms for preventing microbial growth in foods and to ensure food safety (Presser, Ratkowsky, & Ross, 1997).

Numerous studies have reported the approximate pH ranges (Buchanan & Klawitter, 1992; Cole, Jones, & Holyoak, 1990; FDA, 2015; ICMSF, 1980; Presser et al., 1997; Russell & Dombrowski, 1980; Therion, Kistner, & Kornelius, 1982) and temperature ranges (Augustin, Rosso, & Carlier, 2000; Doyle, Mazzotta, Wang, Wiseman, & Scott, 2001; FDA, 2011; ICMSF, 1996; Lund, Baird‐Parker, & Gould, 2000; Nguyen, 2006; Patchett, Watson, Fernandez, & Kroll, 1996) that limit growth of bacterial pathogens.  However, several scientists indicated tolerance of foodborne pathogens for non‐optimal pH and temperature (Gandhi & Chikindas, 2007; Glass, Loeffelholz, Ford, & Doyle, 1992; Shachar & Yaron, 2006), survival of foodborne pathogens in non‐optimal pH and temperature (Rocourt & Cossart, 1997; Zhao & Doyle, 1994), and resistance to the lethal effects of very low pH (Leyer, Wang, & Johnson, 1995). Aligning with those reports, when bacteria are subjected to pH and thermal stresses, sublethal stresses can occur that may lead to differences in terms of their tolerance to thermal process that follows. Bacteria can potentially adapt to non‐optimal pH values and temperatures and may require more time to be destroyed. Semanchek and Golden (1998) reported that variability in the thermal tolerance of *E. coli* O157:H7 strains exposed to different environments affected their resistance to subsequent processes.

Thermal destruction rates are mostly displayed with thermal death decimal reduction time (*D*‐value) and thermal destruction temperature (*z*‐value), and each species of bacteria has its own particular heat tolerance. Therefore, it is essential to determine *D*‐value and *z*‐value to understand and be able to predict the pH and temperature responsiveness of foodborne pathogens during a thermal process. Recognizing the importance of leading foodborne pathogens (*E. coli* O157:H7, *Listeria monocytogenes*,* Salmonella enterica*, and *Staphylococcus aureus*) accountable for the vast majority of foodborne illnesses, hospitalizations, and death in the United States (CDC, 2016), our prior study (C. Kim, R. Alrefaei, M. Bushlaibi, E. Ndegwa, P. Kaseloo, & C. Wynn, unpublished data) evaluated the influence of prior growth temperature (thermal stress) on thermal tolerance of these pathogens. We found that growth temperature clearly influenced the ability of the pathogens to survive subsequent thermal treatments. It has been reported that the exposure of bacterial cells to a previous heat shock provokes an increase in their heat tolerance (Hassani, Cebrián, Mañas, Condón, & Pagán, 2006; Hassani, Condon, & Pagán, 2007; Humphrey, Richardson, Statton, & Rowbury, 1993; Jackson, Hardin, & Acuff, 1996; Katsui, Tsuchido, Takano, & Shibasaki, 1982; Linton, Pierson, & Bishop, 1990; Mackey & Derrick, 1986, 1987a, 1987b; Shenoy & Murano, 1996).

Therefore, the present study was to validate these findings and further elucidate the net effect of pH and temperature on thermal tolerance of *Salmonella* and *Staphylococcus* in vitro, the leading foodborne pathogens accountable for domestically acquired foodborne illness in the United States (CDC, 2016). Due to health concerns, consumers tend to avoid food products with extreme pH either acidic or alkaline but prefer food products with neutral, slightly acidic (~6), or alkaline pH (~9). These ranges of pH are hence chosen in this in vitro study as a foundation for future validation studies involving food matrices. In addition, the terms “cool,” “ambient,” “warm,” and “excessive heat” for 15, 25, 35, and 45°C, respectively, defined as in U.S. Pharmacopeia 659(USP 2017) are used for description purpose of thermal stresses in this article.

## 2 MATERIALS AND METHODS

### 2.1 Bacteria used

Bacterial species used for the study were obtained from American Type Culture Collection (ATCC). Four serovars of *Salmonella enterica* (Enteritidis (ATCC 13076), emerging infectious disease research strain; Montevideo (ATCC 8387), emerging infectious disease research strain; Newport (ATCC 6962), food poisoning isolate; and Typhimurium (ATCC 14028), chicken isolate) and four strains of *Staphylococcus aureus* (ATCC 6538, human lesion isolate; ATCC 29213, human wound isolate; ATCC 33862, enteric research strain; and ATCC 49444, dairy product isolate) were used. Stock cultures of each pathogen strain were maintained in tryptic soy broth (TSB; unless otherwise stated, all media were Bacto, from Becton Dickinson) containing 20% (vol/vol) glycerol (Thermo Scientific) and kept frozen at −80°C. Cultures were transferred three times to TSB supplemented with 0.6% yeast extract (TSBYE; pH 7.4) by loop inoculation at successive 24‐hr intervals and incubated at 35°C before they were used for the study.

### 2.2 Bacterial growth

The pH of the growth medium (TSBYE, pH 7.4) was adjusted using either 1N HCl or NaOH to 6.0 and 9.0. In order to investigate thermal destruction variability in the subsequent heat treatment of foodborne pathogens that induced by pH and thermal stresses during growth in optimum medium (TSBYE), one‐tenth milliliter of each strain was inoculated into 10 ml TSBYE at pH 6.0, 7.4, and 9.0 and incubated for 24 hr at 15°C, 25°C, 35°C, and 45°C, respectively. Following incubation, the bacteria were centrifuged for 10 min at 2,000 ***g*** and 22 ± 2°C in a centrifuge (Model Heraeus Megafuge 16; Thermo Scientific). The pellets were then suspended in 10 ml of sterile 0.85% saline solution and centrifuged again at 2,000 ***g*** for 10 min and resuspended in 10 ml of sterile 0.85% saline solution. Equal volumes of four strains of each bacterial species were mixed to give an inoculum containing approximately equal numbers of cells of each species of *Salmonella* and *Staphylococcus*. In other words, a cocktail containing 4 strains of each bacterial species was used as an inoculum for thermal destruction rate study. Because of difference in bacterial survival/growth rate due to growth temperature and pH, levels of *Salmonella* and *Staphylococcus* obtained after 24‐hr incubation at each temperature and pH are shown in Tables 1 and 2, and used as inocula for thermal destruction study. The inoculum levels shown in Tables 1 and 2 are the average of each initial population of bacteria in triplicates prior to being subjected to thermal treatments at 55, 60, and 65°C. Three independent replicate trials were conducted for thermal treatments at each of slightly acidic (6.0), neutral (7.4), and slightly alkaline (9.0) pH stress condition, and incubation temperature (thermal stress) of 15, 25, 35, and 45°C. Species identity was periodically confirmed on xylose lysine deoxycholate agar (XLD) for *Salmonella* and Baird–Parker agar supplemented with egg yolk tellurite (BP) for *Staphylococcus*. In addition, AOAC‐approved or performance‐tested methods including API 20E and rabbit plasma test were performed.

**Table 1:** The level of *Salmonella* obtained after 24‐hr incubation in TSBYE with pH 6.0, 7.4, and 9.0 at four temperatures (15, 25, 35, and 45°C)*

pH	Incubation temperature (°C)			
	15	25	35	45
6.0	8.4 ± 0.1 A c	9.3 ± 0.2 A a	8.9 ± 0.2 B b	6.4 ± 0.1 B d
7.4	8.3 ± 0.1 A b	9.2 ± 0.2 AB a	9.1 ± 0.2 AB a	6.5 ± 0.0 B c
9.0	8.3 ± 0.1 A c	8.9 ± 0.2 B b	9.3 ± 0.0 A a	6.9 ± 0.0 A d

* Means followed by the same upper‐case letters in the same column within each incubation temperature are not significantly different (*p* > 0.05); means followed by the same lower‐case letters in the same row within the same pH are not significantly different (*p* > 0.05); data represent means ± standard error (*n* = 3).

**Table 2:** The level of *Staphylococcus* obtained after 24‐hr incubation in TSBYE with pH 6.0, 7.4, and 9.0 at four temperatures (15, 25, 35, and 45°C)*

pH	Incubation temperature (°C)			
	15	25	35	45
6.0	7.4 ± 0.4 A b	8.8 ± C 0.0 a	9.0 ± 0.0 A a	6.6 ± 0.0 C c
7.4	7.5 ± 0.2 A b	9.1 ± A 0.0 a	9.0 ± 0.1 A a	7.0 ± 0.2 B c
9.0	6.9 ± 0.1 B c	9.1 ± B 0.0 a	8.7 ± 0.4 A a	7.9 ± 0.2 A b

* Means followed by the same upper‐case letters in the same column within each incubation temperature are not significantly different (*p* > 0.05); means followed by the same lower‐case letters in the same row within the same pH are not significantly different (*p* > 0.05); data represent means ± standard error (*n* = 3).

### 2.3 Thermal destruction

Each bacterial species of inoculum was separately introduced into sterile polyethylene Whirl‐Pak® sample bags (Nasco Fort Atkinson, WI). The bags were 7.5 × 12.5 cm in size with a thickness of 0.057 mm. The sample bags were then completely immersed in a water bath (Lab‐Line Water Bath Model 18,900 AQ; Thermo Scientific) and held at 55°C for 300, 900, and 2,700 s; 60°C for 30, 90, and 270 s; and 65°C for 3, 9, and 27 s. These ranges of temperatures, which are commonly used in cooking beef up to medium rare (Line et al., 1991), are chosen in this study for future validation study in mind on food matrix such as beef or chicken mixed with vegetables. At the end of each exposure time, the sample bags were removed from the water bath and immediately immersed in ice water (0°C) to stop further inactivation due to thermal treatment. Bacterial suspensions in the sample bags were then serially diluted in sterile 0.85% saline solution, surface‐plated on standard method agar (SMA), and incubated at 35°C for 48 hr prior to quantification of bacterial survival. The counts were expressed as log CFU/ml.

### 2.4 Calculation of D‐ and z‐values

The destruction rate curves (R^2^≥0.85) were constructed by plotting the bacterial survivors on the logarithmic scale against the respective exposure time on the linear scale. As described in Redondo‐Solano, Burson, and Thippareddi (2016), the slopes of the thermal destruction rate curves in decimal reduction times (*D*‐values) for *Salmonella* and *Staphylococcus* were calculated from linear regression (inverse of the slope of the regression line) using Excel software (2013, Microsoft) and expressed in minutes. The thermal destruction temperature (*z*‐values) was also calculated by plotting the temperature against log *D*‐value, and the data were fitted by using linear regression with Excel software (2013, Microsoft). The inverse of the slope was reported as the *z*‐value in °C.

### 2.5 Statistical analysis

*D*‐values and *z*‐values for *Salmonella* and *Staphylococcus* were obtained from three independent replications. Data (log CFU per ml, *D*‐values, and *z*‐values) were subjected to an analysis of variance and Duncan's multiple range test (SAS Institute) to determine the significance of the differences (*p* < 0.05) in mean values. Pearson's correlation coefficient was used to evaluate covariance relationships between prior pH and thermal stresses, net effect of pH and thermal stress, and thermal tolerance of bacteria.

## 3 RESULTS AND DISCUSSION

### 3.1 Effect of pH and thermal stress on the level of bacterial inoculum

The effect of pH and thermal stresses, alone and in combination, on the level of *Salmonella* in TSBYE after 24‐hr incubation is shown in Table 1. The levels of *Salmonella* tended to increase with the increment of pH at incubation temperatures of 35 and 45°C, while similar observations were made with the decrement of pH at 15 and 25°C, indicating that slightly alkaline and acidic environment favored bacterial growth at 35 and 45°C, and 15 and 25°C, respectively. A similar increasing phenomenon was also observed for the level of *Staphylococcus* with the increment of pH at 45°C (Table 2). It was noted that the level of both pathogens incubated under excessive heat (45°C) stress was the highest at pH 9.0 among evaluated pH values, indicating that these pathogens may be more resilient to alkaline environment under excessive heat stress. In other words, alkaline food products (e.g., green beans, zucchini, lettuce, sweet potatoes) may be more favorable for the bacteria to grow/survive than acidic and neutral food products when these foods are subjected to excessive heat abuse.

In addition, the overall level of *Salmonella* (8.3. log CFU/ml, average of pH 6.0, 7.4 and 9.0) was significantly (*p* < 0.05) higher than the overall level of *Staphylococcus* (7.3 log CFU/ml) after 24 incubation at 15°C and vice versa at 45°C (6.6 vs. 7.2 log CFU/ml), indicating that *Salmonella* may be more resilient to cool stress than *Staphylococcus* and vice versa to excessive heat. A combination of increment of incubation temperature from 15 to 35°C and pH from 6.0 to 7.4 significantly increased the levels of *Salmonella* (*p* ≤ 0.0027) and *Staphylococcus* (*p* < 0.0001), respectively.

### 3.2 Effect of pH and thermal stress on the thermal tolerance (D‐ and z‐value) of *salmonella*

**Figure 1:**
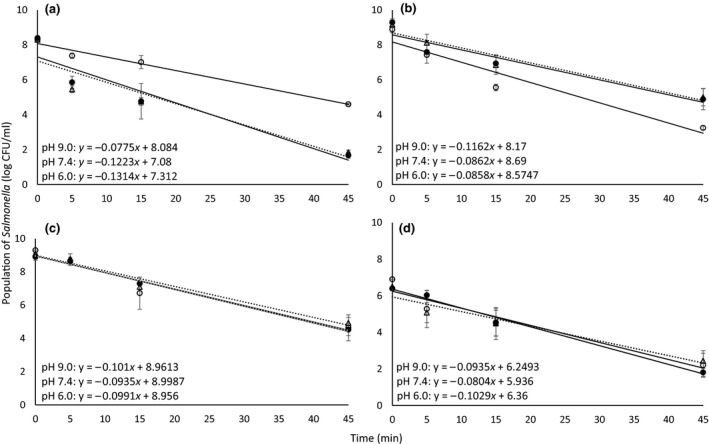
Representative thermal inactivation curves of Salmonella that incubated for 24 hr in TSBYE with pH 6.0 (black circles), 7.4 (white triangles with dashed line), and 9.0 (white circles) at four temperatures (a: 15, b: 25, c: 35, and d: 45°C) and subsequentially subjected to thermal treatments at 55°C. Thermal inactivation gradients are shown in trend line equations in each figure

Based on our previous findings (C. Kim, R. Alrefaei, M. Bushlaibi, E. Ndegwa, P. Kaseloo, & C. Wynn, unpublished data) that thermal stress during bacterial growth influenced the ability of bacteria to survive subsequent thermal treatments, the present study was designed to further evaluate the net effect of pH and thermal stresses on thermal tolerance of *Salmonella* and *Staphylococcus* in vitro. Representative thermal inactivation curves of *Salmonella* that incubated for 24 hr in TSBYE with pH 6.0, 7.4, and 9.0 at four temperatures (15, 25, 35, and 45°C) and subsequentially subjected to thermal treatments at 55°C are shown in Figure 1a–d. The inoculum level (Table 1) at 15°C decreased by 6.6 ± 0.2, 6.4 ± 0.1, and 3.7 ± 0.2 log CFU/ml, respectively, after 45 min of thermal treatment at 55°C (Figure 1a). Thermal inactivation gradients (the magnitude of bacterial population reduction represented as a slope of linear regression) are shown in trend line equations in each figure. Thermal inactivation gradients of the bacteria incubated in pH 6.0 and 7.4 (−0.1314 and −0.1223, respectively) were significantly (*p* < 0.05) higher than those incubated in pH 9.0 (−0.0775). Similar observations were also made for the bacteria subjected to thermal treatments at 60 and 65°C for 270 and 27 s (not shown), respectively, indicating that the bacteria grown in alkaline environment were less susceptible to thermal treatments than those grown in slightly acidic and neutral pH. For the bacteria incubated at 25°C and subjected to thermal treatment at 55°C (Figure 1b), the gradient of thermal inactivation was significantly (*p* < 0.05) higher at pH 9.0 (−0.1162) than that at pH 6.0 (−0.0858) and pH 7.4 (−0.0862). However, pH changes for other incubation temperatures (35 and 45°C) did not show any significant (*p* > 0.05) differences in the gradients of thermal inactivation (Figure 1c,d). *Salmonella* subjected to 15°C and 25°C during overnight incubation at pH 9.0 was the most resistant and susceptible, respectively, to thermal treatment at 55°C than those incubated at pH 6.0 and 7.4. To our knowledge, the present study is the first to report this phenomenon of *Salmonella* in TSBYE response to thermal treatments after overnight incubation at different pH and temperatures. Further research is needed to elucidate the observed differences in the thermal tolerance of the bacteria between cool and ambient temperatures similar to those encountered in food services with storage and holding temperature abuse.

The *D*‐values calculated by fitting the primary log‐linear model to the thermal inactivation curves are shown in Table 3. When the *D*_55°C_ *‐values* of *Salmonella* incubated at 15°C were evaluated, pH 9.0 (13.0 ± 0.6 min) showed the longest, followed by pH 7.4 (8.2 ± 0.3 min) and 6.0 (7.8 ± 0.4 min), indicating that the bacteria under cool stress subjected to alkaline stress were significantly (*p* < 0.05) more resistant to the thermal treatment than those subjected to slightly acidic and neutral pH. This result supports previous work done by Sampathkumar, Khachatourians, and Korber (2004) that pretreatment of *S. enterica* with alkaline solution (pH 10) resulted in a significant increase in thermal tolerance. They reported that the cytoplasmic membrane could play a significant role in the induction of thermal tolerance and resistance to other stresses following alkaline pH treatment.

**Table 3:** *D‐*(min) and *z*‐values (°C) of *Salmonella* that incubated for 24 hr in TSBYE with pH 6.0, 7.4, and 9.0 at four temperatures (15, 25, 35, and 45°C) and subsequentially subjected to thermal treatments at 55, 60, and 65°C*

TTT (°C)	*D‐* and *z*‐value of *Salmonella*/pH and incubation temperature (°C)											
	15			25			35			45		
	6.0	7.4	9.0	6.0	7.4	9.0	6.0	7.4	9.0	6.0	7.4	9.0
55	7.8 ± 0.4 B c	8.2 ± 0.3 B c	13.0 ± 0.6 A a	11.8 ± 0.1 A a	11.9 ± 0.9 A ab	8.8 ± 0.4 B c	10.3 ± 1.2 A b	10.5 ± 1.0 A b	10.1 ± 1.3 A bc	9.7 ± 0.8 B b	12.5 ± 1.7 A a	10.8 ± 1.3 B b
60	0.8 ± 0.1 A c	0.8 ± 0.1 A b	0.9 ± 0.1 A b	1.1 ± 0.1 AB b	0.9 ± 0.1 B ab	1.2 ± 0.2 A a	1.2 ± 0.1 A b	1.2 ± 0.4 A a	1.1 ± 0.1 A ab	1.4 ± 1.2 A a	1.1 ± 0.9 B ab	1.2 ± 0.6 AB a
65	0.2 ± 0.1 A a	0.2 ± 0.1 A a	0.2 ± 0.2 A a	0.2 ± 0.1 A a	0.2 ± 0.1 A a	0.2 ± 0.1 A a	0.3 ± 0.1 A a	0.3 ± 0.1 A a	0.3 ± 0.1 A a	0.2 ± 0.0 A a	0.1 ± 0.1 A a	0.3 ± 0.1 A a
*z*‐value	5.9 ± 0.8 A a	6.4 ± 0.5 A a	5.4 ± 1.1 A a	5.5 ± 0.3 A a	5.7 ± 0.3 A ab	5.8 ± 0.4 A a	6.3 ± 0.6 A a	6.3 ± 0.9 A a	6.2 ± 0.9 A a	5.9 ± 0.3 A a	5.1 ± 0.3 B b	6.1 ± 0.4 A a

* TTT, thermal treatment temperature; means followed by the same upper‐case letters in the same row within each incubation temperature are not significantly different (*p* > 0.05, effect of pH on *D‐* and *z*‐values); means followed by the same lower‐case letters in the same row within the same pH are not significantly different (*p* > 0.05, effect of incubation temperature on *D‐* and *z*‐values); data represent means ± standard error (*n* = 3).

However, *D*‐value for the bacteria incubated at 25°C was the shortest at pH 9 (8.8 ± 0.4 min), followed by pH 6.0 (11.8 ± 0.1 min) and 7.4 (11.9 ± 0.9 min). In other words, the bacteria grown in alkaline environment at 25°C were the most susceptible to the thermal treatment. This contradictory phenomenon of bacterial responsiveness to alkaline environment between 15 and 25°C incubation needs further validation at smaller increment ranges. For incubation temperatures of 35 and 45°C, both decrement and increment of pH from 7.4 to 6.0 and from 7.4 to 9.0, respectively, shortened *D*‐values*,* indicating that decimal reduction time of the bacteria was the longest at pH 7.4. In a similar thermal tolerance study on *Salmonella enterica* grown at 37°C and pH 7, Amado, Vázquez, Guerra, and Pastrana (2014) reported *D*‐values of 0.44–1.35 min at 60°C and 0.22–0.66 min at 65°C, which agree well with the results found in our study (1.1–1.2 min at 60°C and 0.3 min at 65°C) on *Salmonella* grown at 35°C.

Without considering the pH in the growth medium, overall thermal stress from 15 to 45°C during 24‐hr incubation did not significantly (*p* > 0.05) affect the *D*‐values when the bacteria were subjected to the thermal treatments at 55 and 65°C. However, increasing pattern of *D*‐values for the bacteria subjected to the thermal treatment at 60°C was obtained. Although influence of thermal stress on subsequent thermal tolerance of the bacteria was inconsistent at all thermal treatment temperatures evaluated in this study, previous reports (Ingraham, 1987; Mackey & Bratchell, 1989; Mackey & Derrick, 1986; Neidhardt & VanBogelen, 1987; VanBogelen, Acton, & Neidhardt, 1987) demonstrated that exposure to temperatures slightly above the range but nonlethal for normal cell growth leads to progressive loss of bacterial viability and development of bacterial thermal tolerance as a result of heat stress response.

At all thermal treatments, the only net effect (*p* < 0.0001) of thermal and pH stresses on the decrease of *z*‐value was observed with the increment of both thermal stress temperature from 35 to 45°C and pH from 6.0 to 7.4. These results affirm that the bacteria subjected to thermal stress at 35°C under slightly acidic environment (pH 6.0) required the highest increase in temperature to obtain the thermal death decimal reduction time.

### 3.3 Effect of pH and thermal stress on the thermal tolerance of *staphylococcus*

Thermal inactivation data of *Staphylococcus* subjected to thermal treatments at 55, 60, and 65°C following 24‐hr incubation in TSBYE with pH and thermal stresses are presented in Table 4. *D*_55°C_ *‐values* of the bacteria were similar to those observed for *Salmonella,* demonstrating that the bacteria incubated at 15 and 25°C were the longest (12.4 ± 1.2 min) and the shortest (10.9 ± 0.6 min), respectively, at pH 9. At incubation temperatures of 35 and 45°C, decrement and increment of pH from 7.4 to 6.0 and from 7.4 to 9.0, respectively, shortened *D*‐values. Moreover, *D*‐value for the bacteria incubated at pH 9.0 and 35°C and subjected to thermal treatments at 60°C and 65°C was 1.1 ± 0.2 min and 0.2 ± 0.0 min, respectively. These *D*‐values were the shortest among pH ranges evaluated. Without considering the pH, *D*‐value for the bacteria incubated at 45°C and subsequentially subjected to thermal treatment at 55°C was the longest (16.6 ± 3.3 min) and shortest at 60°C (2.2 ± 0.9 min). These findings clearly demonstrated the effect of prior thermal stresses on difference in subsequent thermal tolerance of *Staphylococcus*.

**Table 4:** *D‐*(min) and *z*‐values (°C) of *Staphylococcus* that incubated for 24 hr in TSBYE with pH 6.0, 7.4, and 9.0 at four temperatures (15, 25, 35, and 45°C) and subsequentially subjected to thermal treatments at 55, 60, and 65°C*

TTT (°C)	*D‐* and *z*‐value of *Staphylococcus*/pH and incubation temperature (°C)											
	15			25			35			45		
	6.0	7.4	9.0	6.0	7.4	9.0	6.0	7.4	9.0	6.0	7.4	9.0
55	8.9 ± 0.4 B d	8.8 ± 0.5 B c	12.4 ± 1.2 A ab	17.7 ± 1.3 A a	12.1 ± 0.1 B b	10.9 ± 0.6 B b	12.3 ± 0.8 A c	14.5 ± 1.9 A b	13.2 ± 1.2 A a	15.1 ± 0.8 B b	20.6 ± 1.7 A a	14.0 ± 1.3 B a
60	1.3 ± 0.2 A b	1.4 ± 0.7 A ab	2.0 ± 0.7 A a	1.3 ± 0.1 A b	1.2 ± 0.1 A b	1.2 ± 0.1 A a	2.9 ± 0.4 A a	1.3 ± 0.1 B b	1.1 ± 0.2 B a	2.5 ± 1.2 A ab	2.5 ± 0.9 A a	1.7 ± 0.6 A a
65	0.2 ± 0.1 A b	0.2 ± 0.0 A c	0.2 ± 0.1 A a	0.2 ± 0.1 A b	0.2 ± 0.1 A c	0.2 ± 0.1 A a	0.6 ± 0.1 A a	0.6 ± 0.1 A a	0.2 ± 0.0 B a	0.3 ± 0.0 A b	0.3 ± 0.1 A b	0.3 ± 0.1 A a
*z*‐value	5.6 ± 0.9 A b	5.6 ± 0.4 A b	5.6 ± 0.3 A a	4.8 ± 0.4 A b	5.2 ± 0.6 A b	5.5 ± 0.7 A a	7.6 ± 0.4 A a	6.9 ± 0.6 A a	5.3 ± 0.2 B a	5.7 ± 0.3 A b	5.6 ± 0.3 A b	6.1 ± 0.4 A a

* TTT, thermal treatment temperature; means followed by the same upper‐case letters in the same row within each incubation temperature are not significantly different (*p* > 0.05, effect of pH on *D‐* and *z*‐values); means followed by the same lower‐case letters in the same row within the same pH are not significantly different (*p* > 0.05, effect of incubation temperature on *D‐* and *z*‐values); data represent means ± standard error (*n* = 3).

Increment and decrement of thermal stress temperature from 15 to 35°C (*p* ≤ 0.03) and 45 to 35°C (*p* < 0.001), respectively, significantly increased the *z*‐value*s* of the bacteria with the increment of pH stress from 6.0 to 7.4. The net effect (*p* ≤ 0.03) of thermal and pH stresses on the decrease of *z*‐value was observed in the increment of both thermal stress temperature from 35 to 45°C and pH from 6.0 to 9.0. These results indicate that the bacteria subjected to thermal stress at 35°C required the highest increase in temperature to obtain the thermal death decimal reduction time throughout the pH stresses.

According to the reports compiled by the New Zealand Ministry for Primary Industries (2001) and Albrecht (2017), optimum temperature for the growth of *Staphylococcus* is 37°C. Therefore, the highest *z*‐value (6.6 ± 1.1°C) obtained from our study on the bacteria incubated at 35°C may indicate that within the optimum growth temperature, *Staphylococcus* requires the highest increment of temperature for decimal death reduction. Interestingly, Perl and Schmid (2001) and Lee (2003) reported that most cells respond to a decrease in temperature by inducing a set of cold shock proteins, which play a role in the protection of cells against damage caused by temperature reductions. However, results obtained from our study found no evidence that cool temperature stress (15°C) toughened *Staphylococcus* to subsequent thermal treatment.

When the comparison of thermal tolerance across the species was evaluated, *Staphylococcus* generally displayed significantly (*p* < 0.05) higher *D*‐values than *Salmonella* (Table 5). The observed difference may be due to thermal shock proteins produced by the *Staphylococcus*. Cordwell, Larsen, Cole, and Walsh (2002) and Stewart (2003) reported that although the ability to produce and intensity of production may vary from strain to strain, *Staphylococcus aureus* are known to produce thermal shock proteins and have a relatively high heat resistance. It was also noted that *Staphylococcus* incubated at pH 6 and 35°C demonstrated higher *D‐* and *z*‐value*s* than *Salmonella* indicating its longer thermal tolerance. Other studies (Jay, 2012; Knabel, 1989; Mai‐Prochnow, Clauson, Hong, & Murphy, 2016; Sun, 2012) reported that gram‐positive bacteria (e.g., *Staphylococcus*) are relatively more heat resistant than gram‐negative bacteria (e.g., *Salmonella*) due to the difference in the structure of their cell wall.

**Table 5:** Summary of *D‐* (min) and *z*‐value*s* (°C) of *Salmonella* and *Staphylococcus* that incubated in TSBYE with pH 6.0, 7.4, and 9.0 at four temperatures (15, 25, 35, and 45°C) for 24 hr and subsequentially subjected to thermal treatments at 55, 60, and 65°C*

TTT (°C)	*D‐* and *z‐value* of bacteria/pH and incubation temperature (°C)											
	15						25					
	6.0		7.4		9.0		6.0		7.4		9.0	
	SM	SA	SM	SA	SM	SA	SM	SA	SM	SA	SM	SA
55	7.8 b	8.9 a	8.2 a	8.8 a	13.0 a	12.4 a	11.8 b	17.7 a	11.9 a	12.1 a	8.8 b	10.9 a
60	0.8 b	1.3 a	0.8 a	1.4 a	0.9 b	2.0 a	1.1 a	1.3 a	0.9 b	1.2 a	1.2 a	1.2 a
65	0.2 a	0.2 a	0.2 a	0.2 a	0.2 a	0.2 a	0.2 a	0.2 a	0.2 a	0.2 a	0.2 a	0.2 a
*z‐value*	5.9 a	5.6 a	6.4 a	5.6 a	5.4 a	5.6 a	5.5 a	4.8 a	5.7 a	5.2 a	5.8 a	5.5 a

	35						45					
	6.0		7.4		9.0		6.0		7.4		9.0	
	SM	SA	SM	SA	SM	SA	SM	SA	SM	SA	SM	SA
55	10.3 a	12.3 a	10.5 b	14.5 a	10.1 b	13.2 a	9.7 b	15.1 a	12.5 b	20.6 a	10.8 b	14.0 a
60	1.2 b	2.9 a	1.2 a	1.3 a	1.1 a	1.1 a	1.4 a	2.5 a	1.1 a	2.5 a	1.2 a	1.7 a
65	0.3 b	0.6 a	0.3 b	0.6 a	0.3 a	0.2 a	0.2 b	0.3 a	0.1 b	0.3 a	0.3 a	0.3 a
*z‐value*	6.3 b	7.6 a	6.3 a	6.9 a	6.2 a	5.3 a	5.9 a	5.7 a	5.1 a	5.6 a	6.1 a	6.1 a

* TTT, thermal treatment temperature; SM, *Salmonella*; SA, *Staphylococcus*; means followed by the same lower‐case letters in the same row within each incubation temperature and pH are not significantly different (*p* > 0.05).

In general, findings from our study agree with the results presented by others (Jackson et al., 1996; Kaur, Ledward, Park, & Robson, 1998; Semanchek & Golden, 1998) that heat tolerance of bacteria tends to be greater when cells were grown at elevated temperatures (e.g., 37 or 40°C vs. 10 or 25°C). This phenomenon was illustrated by Katsui, Tsuchido, Takano, and Shibasaki (1981) that increased heat tolerance associated with changes in growth temperature was attributed to alteration of the fatty acid composition in bacterial membranes. Beuchat (1978) postulated that bacteria grown at low temperatures may incorporate more unsaturated fatty acids into their cell membranes in order to maintain functional membrane fluidity. Therefore, decreased heat tolerance may occur due to the reduced melting point of unsaturated fatty acids within the cell membrane. Other scientist have also indicated that the stress response of bacteria to sublethal environmental stresses such as changes in temperature, starvation, or high osmolarity may provide cross‐protection to a variety of postphysical and postchemical stresses including heat and acid (Allen, Lepp, McKellar, & Griffiths, 2010; Arnold & Kaspar, 1995; Erdoğrul, Erbilir, & Toroğlu, 2006; House et al., 2009; Jeong, Baumler, & Kaspar, 2006; Lange & Hengge‐Aronis, 1994; Leenanon & Drake, 2001; Nair & Finkel, 2004). Numerous studies reported that the exposure of bacterial cells to a previous heat shock provokes an increase in their heat tolerance which may have important practical consequences, such as the survival of microorganism to the treatment applied (Hassani et al., 2006, 2007; Humphrey et al., 1993; Jørgensen, Panaretou, Stephens, & Knøchel, 1996; Katsui et al., 1982; Linton et al., 1990; Mackey & Derrick, 1986, 1987a, 1987b; Shenoy & Murano, 1996).

## 4 CONCLUSION

Although the effect of pH stress alone during the incubation on the thermal tolerance of both bacteria was generally insignificant, increasing pattern of thermal death decimal reduction time was observed with the increment of incubation temperature. Both bacteria incubated at optimum growth temperature (35°C) required the highest temperature increase to reduce the thermal death decimal reduction time*. Staphylococcus* generally displayed higher tolerance to thermal treatment than *Salmonella*. Findings from our prior (unpublished data) and current study clearly demonstrated the relative changes in time–temperature profiles for the destruction of the pathogens tested and will help inform decisions about the stringency of environments needed for the proper intervention of foodborne pathogens. While much can be learned from the findings in this study, additional research efforts are needed to validate the differences in thermal tolerance of the tested bacteria in food matrices and also the effect of variety of other food processing‐related stresses (i.e., acid, fat, protein, starch, sugar, and water) in vitro and in situ.

## CONFLICT OF INTEREST

The authors declare no conflict of interests.

## ETHICAL STATEMENT

This study does not involve any human or animal testing.
